# Procedures to Select Digital Sensing Technologies for Passive Data Collection With Children and Their Caregivers: Qualitative Cultural Assessment in South Africa and Nepal

**DOI:** 10.2196/12366

**Published:** 2019-01-16

**Authors:** Brandon A Kohrt, Sauharda Rai, Khanya Vilakazi, Kiran Thapa, Anvita Bhardwaj, Alastair van Heerden

**Affiliations:** 1 Division of Global Mental Health Department of Psychiatry and Behavioral Sciences George Washington School of Medicine and Health Sciences Washington, DC United States; 2 Department of Global Health Milken School of Public Health George Washington University Washington, DC United States; 3 Research Department Transcultural Psychosocial Organization Nepal Kathmandu Nepal; 4 Jackson School of International Studies University of Washington Seattle, WA United States; 5 Research Department Human Sciences Research Council Pietermaritzburg South Africa; 6 Department of Health Policy and Management, College of Public Health University of Georgia Athens, GA United States; 7 Department of Population, Family and Reproductive Health Bloomberg School of Public Health Johns Hopkins University Baltimore, MD United States; 8 Human and Social Development Human Sciences Research Council Pietermaritzburg South Africa; 9 Medical Research Council/Wits Developmental Pathways for Health Research Unit Department of Paediatrics, Faculty of Health Science University of the Witwatersrand Johannesburg South Africa

**Keywords:** child development, confidentiality, culturally competent care, developing countries, global health, mental health, mobile phones, wireless technology

## Abstract

**Background:**

Populations in low-resource settings with high childhood morbidity and mortality increasingly are being selected as beneficiaries for interventions using passive sensing data collection through digital technologies. However, these populations often have limited familiarity with the processes and implications of passive data collection. Therefore, methods are needed to identify cultural norms and family preferences influencing the uptake of new technologies.

**Objective:**

Before introducing a new device or a passive data collection approach, it is important to determine what will be culturally acceptable and feasible. The objective of this study was to develop a systematic approach to determine acceptability and perceived utility of potential passive data collection technologies to inform selection and piloting of a device. To achieve this, we developed the *Qualitative Cultural Assessment of Passive Data collection Technology* (QualCAPDT). This approach is built upon structured elicitation tasks used in cultural anthropology.

**Methods:**

We piloted QualCAPDT using focus group discussions (FGDs), video demonstrations of simulated technology use, attribute rating with anchoring vignettes, and card ranking procedures. The procedure was used to select passive sensing technologies to evaluate child development and caregiver mental health in KwaZulu-Natal, South Africa, and Kathmandu, Nepal. Videos were produced in South Africa and Nepal to demonstrate the technologies and their potential local application. Structured elicitation tasks were administered in FGDs after showing the videos. Using QualCAPDT, we evaluated the following 5 technologies: home-based video recording, mobile device capture of audio, a wearable time-lapse camera attached to the child, proximity detection through a wearable passive Bluetooth beacon attached to the child, and an indoor environmental sensor measuring air quality.

**Results:**

In South Africa, 38 community health workers, health organization leaders, and caregivers participated in interviews and FGDs with structured elicitation tasks. We refined the procedure after South Africa to make the process more accessible for low-literacy populations in Nepal. In addition, the refined procedure reduced misconceptions about the tools being evaluated. In Nepal, 69 community health workers and caregivers participated in a refined QualCAPDT. In both countries, the child’s wearable time-lapse camera achieved many of the target attributes. Participants in Nepal also highly ranked a home-based environmental sensor and a proximity beacon worn by the child.

**Conclusions:**

The QualCAPDT procedure can be used to identify community norms and preferences to facilitate the selection of potential passive data collection strategies and devices. QualCAPDT is an important first step before selecting devices and piloting passive data collection in a community. It is especially important for work with caregivers and young children for whom cultural beliefs and shared family environments strongly determine behavior and potential uptake of new technology.

## Introduction

### Background

The recognition of early child development as a domain of global importance and the inclusion of specific child development indicators in both the Sustainable Development Goals (SDGs) [1] and the United Nations Secretary General’s Global Strategy for Women’s, Children’s and Adolescents’ Health [2] has refocused attention on a life-course perspective and the need to ensure that children meet their developmental potential. Assessing children’s developmental progress and caregiver-child relationships longitudinally is expensive and time-consuming, with few existing, valid methods available to effectively measure and monitor at a population level [3]. Paper-based tools require careful quality assurance and quality control review, which double data entry time burden. Traditional approaches to audiovisual recordings can be equally time consuming because of required transcription and structured coding. These existing limitations point to the potential gap that could be bridged by using newer technology.

The use of mobile technologies to leverage their capabilities and functionality to support public health care is called mobile Health (mHealth) [4]. mHealth in low- and middle-income countries (LMICs) first started with strengthening the data system on computer-based platforms but has transcended to data collection, training, facilitated communication among health workers, decision support, supervision, and health promotion in recent years [5,6].

Among the first steps to transform mHealth potential into transformative public health impact is the United States’ National Institutes of Health’s toolbox. The toolbox comprises a validated set of freely available measures that can be used to quickly (within 2 hours or fewer) assess cognitive, sensory, motor, and emotional function in a diverse range of contexts [7]. All measures are available electronically for use on an iPad. Rather than requiring highly trained research staff to simultaneously monitor time, record responses, and interact with the child, these electronic assessments simplify test administration and reduce cognitive load, thereby improving data accuracy.

As technology advances, new avenues of exploration in the application of technology to early child development are emerging. The Language Environment Analysis (LENA) system is a comprehensive assessment of the home linguistic environment of infants and children. The LENA system consists of a small child-safe recorder that is worn, in a comfortable custom-designed vest, by the child during the day. Recordings are translated into data about the linguistic environment, which can be viewed and analyzed using specialist software [8]. Other developmental uses include tools for enhanced early education, for example, educational magic toys, which make use of augmented reality technology [9,10]. The growing array of baby wearables that track heart rate, movement, breathing, and other physiological measures also holds promise for improving child health research and its public health impact in LMIC [11-14].

Although the toolbox and other app versions of psychometric tests are well described [7,15,16], little has been written about how other mother and child assessment methodologies can benefit in a similar way from technological progress. Naturalistic observation, caregiver and clinical interviews, direct assessment, and coding of interactions are still primarily the global tools of choice. The challenge is that many of the issues of interest are best examined through methods that require manual collection and coding of unstructured data. For example, caregiver-child attachment is time-consuming to code and requires high levels of training and honed skills for coding the observational audio-visual recordings. Such demands are barriers to scaling up the method for widespread use.

Accurately and reliably measuring child development to track progress toward meeting the SDGs requires a suite of research tools that go beyond mobile apps and are acceptable, confidential, safe, nondisruptive, and have utility. One avenue of exploration is passively collecting, transforming, and analyzing data generated by mobile phones, wearables, and other small sensors that can be embedded in the environment [17]. Advancements in the fields of digital sensors, computation, storage, and communications have turned mobile phones into powerful mobile sensing devices [18]. Among the sensors included in modern mobile phones are accelerometer, altimeter, digital camera, microphone, Global Positioning System (GPS), Bluetooth proximity, and oximeter among others [19].

Analyzing the digital traces produced by these sensors could enable the assessment, detection, and monitoring of key developmental processes in an automated, expeditious, and scalable manner. Although single-sensor–based systems are useful, they are often limited in both accuracy and the number of behavioral activities that can be tracked. To counteract this limitation, multisensor systems are now preferred [18]. Using the ecological systems theory as a foundation [20], Table 1 presents an overview of the types of sensors and information that could be obtained about a child’s world through the collection and analysis of these passive data producing sensors.

Although it may technically be feasible in certain contexts to collect these diverse data sources, acceptability will vary due to, among other things, the intrusiveness of the approach. User-centered studies suggest that technologies intended for intimate personal use need to comply with a number of factors if they are to be deemed usable and acceptable and sustain user engagement [21]. Taking into account both human and technical aspects, 6 factors have been found to influence adoption [22]; they include (1) supporting fundamental human needs such physiological and safety needs; (2) cognitive load—ease of use, perceived risks, and fears; (3) social factors—privacy, cultural acceptance, and influence over social interactions; (4) physical aspects—device size, conform, and appearance; (5) participant demographics—age, gender, and culture influence preferences and perceptions toward devices; and (6) technical expertise of the user—with more expertise increasing confidence and use.

These principles inform a growing body of evidence for the feasibility and usefulness of sensor devices in maternal and child research. Cameras and the PhotoVoice methodology have, for example, been used with children aged as young as 3 years to gain a better understanding of how they perceive their community, relate to being orphaned, and understand their infection with HIV [23,24]. First-person photography, through body-worn cameras, has also shown promise as a novel methodology for capturing the world through the eyes and perspective of the child [25].

The work of Mehl [26], whose electronically activated recorder yields valuable acoustic logs of people’s day-to-day experience, is an example of how audio data generated from a microphone could give valuable insight into a child’s life. The LENA system builds on this idea by using a small audio recorder, worn by the child through the day, to periodically sample the auditory environment. These audio data are loaded into the LENA system and are immediately translated into information about the environment of the child [27].

Radio-Frequency Identification Devices (RFID) is the primary approach used so far to track interpersonal proximity. RFID tags are activated in the presence of a receiver and can generate a small radio frequency that is recorded by the receiver. For example, an RFID-based system was implemented to track the spread of hospital-acquired infections in the pediatric ward [28]. The same RFID system was used in France to better understand face-to-face contact between students to better understand how these interactions shaped social networks and facilitated the propagation of infectious disease [29].

One limitation of the current literature base is that most of these studies are currently being produced in high-income countries in controlled laboratory or hospital settings. Furthermore, many do not provide a method for establishing participants’ understanding of technology and the data that can be collected and mined from these devices.

### Objective

This study, therefore, aims to develop a systematic method to inform understanding of how families and caregivers from LMICs perceive the suitability and acceptability of a range of passive digital data collecting sensors that could be incorporated into the home environment. The findings from this approach should be able to directly inform selection of devices and passive data collection strategies with the greatest likelihood of adoption for home-based interventions. We refer to our proposed systematic approach as the *Qualitative Cultural Assessment of Passive Data collection Technology* (QualCAPDT).

**Table 1:** Passive digital sensors and an example of the type of information they could produce at each level of ecological systems theory.

Ecological level	Activity	Sensors	Information
Individual (child)	Movement	Accelerometer, altimeter, gyroscope, and GPS^a^	Activities of daily living
Individual (child)	Physiology	Electrocardiogram, electromyograph, electroencephalogram, electrodemograph, oximeter, and thermometer	Assessment of toxic stress
Microsystem (peers, family, and caregiver)	Interaction	Wi-Fi proximity, Bluetooth proximity, microphone, digital camera, and digital video	Nurturing care
Exosystem (neighborhood, mass media, and extended family)	Environment	Digital camera, digital video, and environmental sensor	Air, noise, and water pollution
Macrosystem (culture, social conditions, and economic system)	Human development	Microphone	Language development and exposure to cultural practices

^a^GPS: Global Positioning System.

## Methods

### Settings

Research was conducted in South Africa and Nepal. These countries are ideal sites to develop a procedure that can be used to select devices and data collection approaches that will be acceptable and feasible in settings with low-literacy populations unfamiliar with passive sensing technology. The countries have high rates of poverty and childhood morbidity and mortality with limited access to specialized child health and mental health services. Moreover, the countries are exemplified by large health disparities and poor outcomes with traditional health delivery approaches, thus necessitating greater use of technology.

Moreover, there are preliminary successes for mHealth in these settings. In South Africa, mHealth is increasingly used for HIV/AIDS prevention and care, including home-based testing and counseling to promote treatment engagement and adherence [30,31]. Text messaging through mobile phones has been used to collect feedback on maternal-child health services throughout South Africa and resolve areas of poor-quality care [32]. mHealth in Nepal has worked in different capacities such as improving communication and coordination between health workers in rural areas and district hospitals, strengthening community-based surveillance systems, and improving maternal and neonatal health outcomes [33,34]. Furthermore, mHealth has been successful in achieving targets for reduced maternal mortality through increased health facility attendance and institutional delivery in Nepal [35]. However, in both countries, passive sensing data collection has received limited attention and would benefit from qualitative exploration before selecting and piloting new approaches. Furthermore, methods used to determine cultural acceptability and feasibility before selecting devices for pilot could be of great benefit throughout LMICs, other low-resource settings, and a context with diverse cultural groups.

In South Africa, the study was conducted in the Sweetwaters region of the Greater Edendale Area of Pietermaritzburg, KwaZulu-Natal. This location has been the site of ongoing research on public health initiatives led by the Human Sciences Research Council (HSRC). The Sweetwaters area is emblematic of rural regions in South Africa that have suffered high rates of maternal HIV and mother-to-child transmission. However, through public health programs, these rates have dramatically reduced over the past decade. In Nepal, the study was conducted in Sankhu, Manamaiju, and Phutung, which are all located approximately 30 to 60 min away from Kathmandu. These areas were heavily affected by the 2015 Nepal earthquake. Sankhu was devastated with more than 100 persons killed on the day of the first earthquake. Sankhu, although having access to Kathmandu, lacks child health specialty services, and there are no local mental health services. Manamaiju was comparatively closer to the city but did not have mental health services in their local health posts. Research in these sites was conducted in 2016 to 2017.

### Qualitative Cultural Assessment of Passive Data Collection Technology

We developed the QualCAPDT procedure by adapting methods commonly used in cultural anthropology [36,37]. We had 2 objectives in the development of QualCAPDT. The first was to have a systematic process that could be replicated in other settings to evaluate the acceptability and perceived utility of different passive data collection strategies before development or adaptation of the technology. The goal was to avoid selection of data collection platforms before receiving input from end-user communities, which would lead to potential waste of resources for technologies that would not be adopted. The second was to gain information about how participants viewed, among other issues, the ethical risks they would be exposed to by participating in a study that collected passive digital data about their behavior in their homes. A crucial outcome was to protect people’s ethical rights in research because passive data collection of daily family life is an invasive process. Our hope was that QualCAPDT would provide insight into privacy, confidentiality, and the major ethical responsibilities researchers have in this new technological age of health interventions.

To frame our 2 objectives, we used the following domains, adapted from Buenaflor and Kim’s 6 human factors [22], to guide all participant discussions about the devices and data under review:

- Domain 1: *Confidentiality* referring to the degree to which the device would protect personal information; this was an ethical domain we added to the 6 human factors.
- Domain 2: *Safety* referring to concerns that the device would pose health risks or put a child or family at the risk of mugging or theft; this domain captures Buenaflor and Kim’s first human factor—safety.
- Domain 3: *Social acceptability* referring to the degree to which family members and neighbors may have negative responses to introduction of the device; this reflects the third human factor—social factors—and human factor 5—demographic perceptions.
- Domain 4: *Noninterference* referring to the degree to which the device would negatively impact physical functioning, activities, or daily routines; this reflects the fourth human factor—physical aspects.
- Domain 5: *Utility* referring to the perceived benefit of the device for improving caregiver and child health, development, and mental health; this incorporates aspects of human factor 2—ease of use—and human factor 6—technical expertise.

#### Step 1: Development of Videos

Narrative focus group discussions (FGDs) are a technique commonly used in cultural anthropology and public health [38-40]. In narrative FGDs, participants are typically read a story or scenario at the beginning of the session, then they comment according to probes provided by the facilitator. Given the lack of familiarity with passive data collection devices among community health workers and caregivers in rural South Africa and Nepal, we felt that narratives about the devices would have been insufficient for participants to understand the technology and types of data capture. Therefore, videos ranging in length from 3 to 6 min were produced, demonstrating the technologies in the local settings.

In South Africa, the production of the videos was conducted by a group of research assistants over a period of a month. In Nepal, the video production task was assigned to a team of independent contractors, and they took around 1 month to produce the final cuts. Five videos, each demonstrating a technology, were produced in both countries. Separate videos were produced for South Africa and Nepal so that participants could relate to the experiences of persons in the video. Videos were in the local languages of the participants, isiZulu with English subtitles in South Africa and Nepali in Nepal.

**Figure 1:**
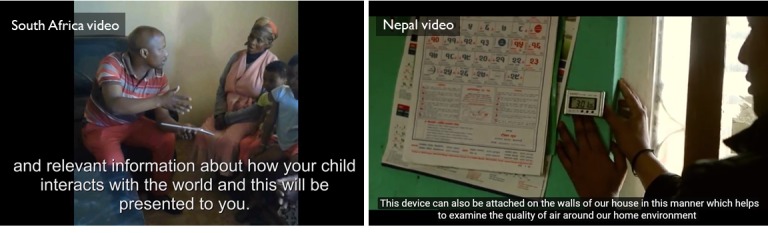
Screenshots from videos demonstrating passive data collection devices in South Africa and Nepal.

The video content was similar between the 2 countries. The videos provided images of the technology, a scenario in which a researcher explains the technology to a rural family with a small child, and, when possible, an example of the output of the technology that could be used for health interventions. In Nepal, the video was prefaced with information on the role of female community health volunteers (FCHVs) as these would be the individuals managing the devices. Screenshots from the South Africa and Nepal videos are provided in Figure 1. Individual videos are indexed below as Multimedia Appendices.

#### Step 2a: Conduct Video Focus Group Discussions

**Figure 2:**
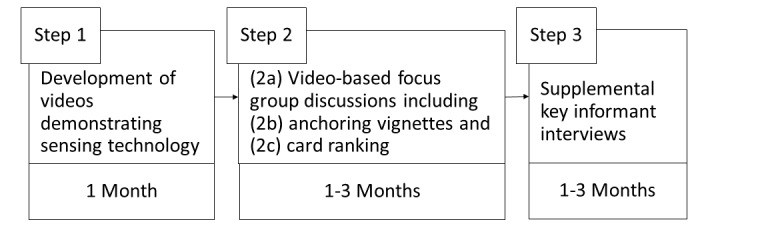
Qualitative Cultural Assessment of Passive Data collection Technology (QualCAPDT) process timeline of piloting in South Africa and Nepal.

After the videos were produced, they were integrated into the FGDs with community health workers and caregivers (see Figure 2 for timeline). FGDs were conducted in isiZulu in South Africa and Nepali in Nepal. In both countries, the FGDs for community health workers began with general questions about health needs of children and their daily routines, including any health and development concerns that were common in that community. The FGDs then transitioned to showing videos. After the videos, the groups rated attributes using anchoring vignettes (step 2b) followed by card ranking tasks (step 2c), and then, supplemental interviews were conducted with other stakeholders (step 3).

In South Africa, the FGDs were organized through a community outreach team that works and lives within the community where the participants were sampled. An equivalent of US $5 was issued out to each participant as reimbursement for the time that they spent in the FGD. The FGDs were conducted within the community in a central community hall. All FDGs were conducted by qualitative interviewers using IsiZulu as a medium of communication. The videos were projected onto a wall using a projection system connected to a laptop. The 4 FGDs took a period of 9 days for completion due to the availability of both the community hall and the participants in these groups.

In Nepal, the FGDs were scheduled with the help of FCHV. One of the FCHV was contacted by phone 2 days before the FGD and were asked to gather participants for FGDs. The participants were provided nonmonetary compensation, for example, household items such as soap, toothpaste, brush, and lunch, for their time and effort. For FCHV FGDs, the data were collected in a quiet office room in a health care facility. Caregiver FGDs took place in 1 of the caregiver’s homes. One of the authors (KT) moderated the discussions along with a research assistant who did note-taking. The discussions were held in the local language. We also audio recorded the FGDs after receiving consent from all the participants. The FGD guide was semistructured. The videos were shown in a laptop where participants were seated just in front so that everyone could see and hear what was happening in the video. In addition to the use of anchoring vignettes (described below), several probes were used during the FGDs to elicit responses from different participants.

Some examples of follow-up questions post video including probes used in both sites are shown below:

- What do you think of the device? (PROBE: Have you seen such device before? How do you like the device overall?)
- What do you think are the barriers to using this device? (PROBE: Electricity problems, incite arguments in a family, etc)
- Can you think of any example of how this device is useful? (PROBE: What information could this device collect, and how can you use that information?)
- How feasible do you think it is to use these devices in your community? (PROBE: How many people in your community use mobile phones regularly? What cultural practices might hinder the use of the device? Do you think there is risk of theft or breakage of the devices?)

#### Step 2b: Rating of Preferences Through Anchoring Vignettes

**Figure 3:**
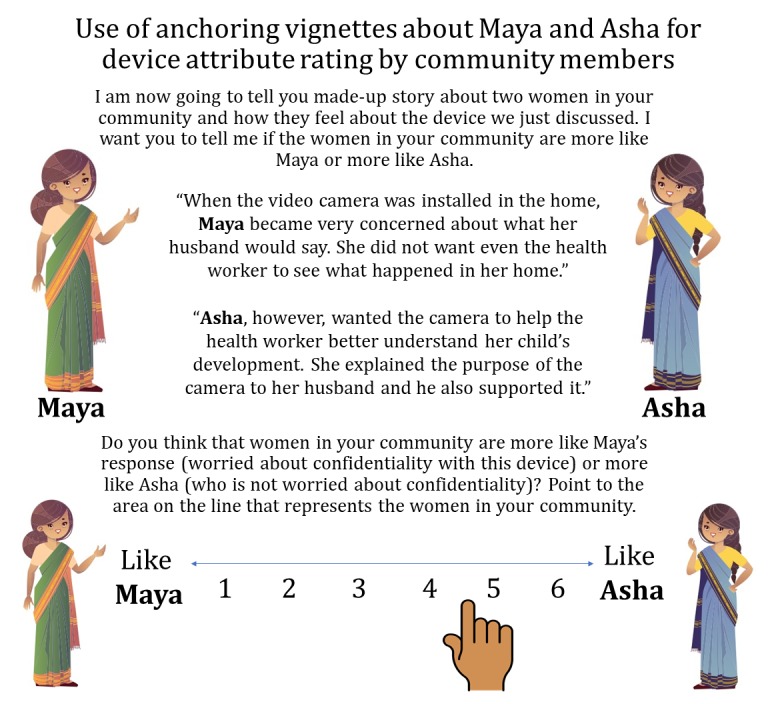
Anchoring vignette elicitation technique to rate devices by attribute domains. (The anchoring vignettes, including all data collection materials and videos, were presented in the local language of participants: isiZulu in Kwa-Zulu Natal, South Africa, and Nepali in Kathmandu Valley, Nepal. Names and illustrations should be adapted to local cultural context).

Anchoring vignettes are a structured elicitation tool commonly used in anthropological research, health behavior studies, and public opinion polls [41-43]. As individuals may have a social desirability bias toward responding in the affirmative, anchoring vignettes can be used to normalize a range of responses [44]. When initially piloting preferences for the devices in this study (unpublished), we found that few women had negative responses. Therefore, we created 2 vignettes to anchor the response options. For each criterion, there was 1 vignette that ranked the device high on the criterion and 1 vignette that ranked the device low on the criterion. For example, in Nepal, we referred to Maya and Asha, with Maya vignettes having a concern about the device and Asha being supportive about the device on that attribute. Then, participants were asked to say whether they felt that women in their community were more likely to be like Maya or Asha (see Figure 3). The anchoring vignettes were presented in isiZulu in South Africa and in Nepali in Nepal.

When doing the rating according to the anchoring vignettes, participants are encouraged to describe their thought process and discuss as a group why they are making certain decisions. This process is based on techniques from anthropological research on cultural domain analysis [45]. In this process, participants prompted to describe *attributes* that lead to categorizing in a certain way. For example, what are the *attributes* of a device that lead to it being categorized more closely to Maya’s perspective in one domain but then closer to Asha’s perspective in another domain. This type of prompting reduces the likelihood that a device is ranked all toward Maya on every domain or all toward on Asha on every domain. Through this prompting, the participants consider each domain independent of the others and identify the attributes that contribute to the device’s categorization in each domain. This is rich qualitative information, which often came in the form of participants debating the ways in which community members think more like Maya or Asha.

#### Step 2c: Card Ranking Task

After rating with anchoring vignettes, we used a card sort ranking task at the end of the FGD [36]. In this activity, each device had a unique card with a photograph of the device, and participants were asked to sort those cards according to each of the attributes. For example, the 6 devices were ranked in order from most to least confidentiality, and similarly, all devices were ranked from the most to least useful for child health promotion. This is a forced-choice approach in which participants have to make cognitive decisions to up- or down-rank certain devices. During this process, participants are encouraged to describe the thought process and decision making that influences their ranking. The cards were images of the devices and did not include written language. Discussions during the card ranking were conducted in isiZulu in South Africa and in Nepali in Nepal.

Example prompts and probes during the card ranking task were as follows:

- Example probe for confidentiality: Among all the devices we just discussed, which device do you think protects most of your personal information? And next? And next?
- Example probe for safety: Of all the devices, which one do you think can be safely placed anywhere at home and will not be prone to breakage or theft? And next? And next?
- Example probe for acceptability: Of all the devices, which one do you think will be most accepted by you and your family members? And next? And next?
- Example probe for noninterference: Of all the devices, which one do you think most hinders with your daily activities? And next? And next?

#### Step 3: Use of Supplemental Interviews and Data Collection

In addition to the FGDs structured around the videos with anchoring vignettes and card sort ranking, we conducted additional key informant interviews to explore other themes that would reflect community norms, preferences, and perceived utility and feasibility of the devices. In some of these individual interviews, the videos were also used to elicit discussion.

Example questions from the supplemental interviews were as follows:

- How would families feel if you collected information about conversations between family members using these technologies?
- What information could be collected around mealtime activities?
- What are the main times of day when caregivers and children are together, and what information could be collected then?
- Whose permission do we need to keep this device at home?

In South Africa, the supplementary interviews were conducted with local organizations that work in the early childhood development sphere. The participants were recommended by the South African principal investigator who had extensive experience working in the area or by research assistant based on collaborating organizations working in the area of child health and/or mobile technology. These participants were not reimbursed for their time because they were professionals often collaborating in research and health initiatives in the area. The participants were contacted through the community outreach team for available times when the interview could be conducted. Supplemental interviews in South Africa were conducted in English because they were often with organizational staff educated in English who were South African but may not have been native to Sweetwaters.

In Nepal, for FCHV interviews, the contact information of the FCHV was obtained from local health facility. They were contacted 2 days before the day of data collection and scheduled the time. For caregivers’ interviews, we obtained the contact information from FCHV. The interviewees were provided nonmonetary compensation as with the FGDs, for example, household items such as soap, toothpaste, and brush, for their time and effort. The data were collected in a quiet room in a FCHV or caregiver’s home. One of the authors (KT) and 2 research assistants were involved in conducting interviews. We also audio recorded the entire interview after written consent from the interviewee. The interview guide was semistructured. When videos were shown, a laptop was used. The information generated from interviews differed from FGDs in that interviews helped us to identify attitudes and perceptions of individuals in depth as opposed to coming to a general consensus in FGDs. In addition, the interviews may have also differed from other standard semistructured interviews in that participants watched videos of technologies without group discussions and the use of multiple probe questions. Supplemental interviews were all conducted in Nepali.

### Passive Data Collection Devices Evaluated

We used the QualCAPDT method to assess the suitability and acceptability of 8 approaches to collecting passive digital sensor data from 5 devices about caregivers and children in the home. They ranged from invasive approaches that produce rich data to less-invasive approaches that result in less rich data.

#### Device 1: Video Recorder (Two Approaches)

There were 2 forms of video recording presented to participants. One was a continuous recording with a camera mounted in the living room of the home. Families were shown that data would be stored on secure digital cards that would be removed and reviewed by the researcher together with the family. The participants were also told that a time-lapse version was possible in which video would be captured every 15 min for 30 seconds. The participants were told that neither data collection platform would record sound. The South African video demonstrating video recording is available in Multimedia Appendix 1 and the video from Nepal is provided in Multimedia Appendix 2.

#### Device 2: Audio Recorder (Two Approaches)

Similarly, participants were presented with technology that could collect audio from the home environment. It was explained to participants that the technology could be set to record continuous or episodic audio, with an example of the latter being recordings made every 15 min for 30 seconds. The videos demonstrated a fixed recording device in South Africa (Multimedia Appendix 3) and a mobile recording device in Nepal (Multimedia Appendix 4).

#### Device 3: Wearable Camera (One Approach)

**Figure 4:**
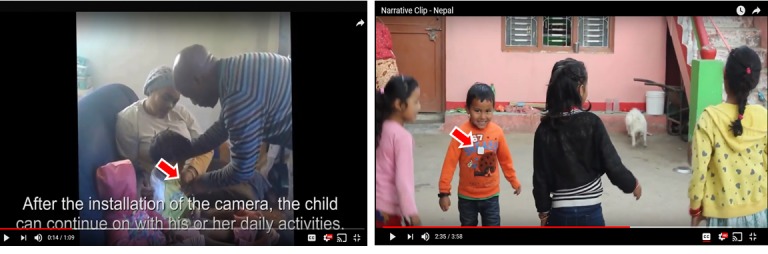
Screenshots of videos demonstrating wearable time-lapse camera for children. Red arrows point toward the wearable device on the child.

A wearable time-lapse camera was demonstrated as a technology that could be used to capture images from the child’s point of view. Videos demonstrated how children could wear the devices (see screenshots in Figure 4). The images captured on the devices in the children’s daily life were presented in the video. The South Africa wearable camera video is provided in Multimedia Appendix 5, and the video from Nepal is provided in Multimedia Appendix 6.

#### Device 4: Bluetooth Beacon (One Approach)

A Bluetooth beacon was displayed in a video as a way to evaluate when the target caregiver and target child are in close proximity. This was illustrated through a small coin size plastic toy that could be attached to the child and that sent out a signal that could be received by a mobile phone carried by the caregiver. A mock output was shown in the video to simulate data that could be reported on time the caregiver and child spend together. The video demonstration for South Africa is provided in Multimedia Appendix 7, and the video demonstration for Nepal is provided in Multimedia Appendix 8.

#### Device 5: Environmental Sensor (One Approach)

Finally, a room-based environmental sensor was presented as a technology that could report on temperature, humidity, and air quality within the home. The devices were fixed in the home, and families were told they could be placed in any room in the household that they preferred to assess air quality. The video demonstrating the environmental sensor in South Africa is provided in Multimedia Appendix 9, and the video for Nepal is provided in Multimedia Appendix 10.

### Participant Recruitment

In South Africa, 38 participants were recruited for 4 FGDs. Participants included health organization workers, community health workers, and caregivers. Caregivers were deemed eligible if they had a child in their care who resided with them in the household. Of the 4 FGDs conducted, 2 FGDs were with health organization workers and community caregivers (n=17) and 2 were with caregivers (n=21).

In Nepal, 69 participants were recruited. There were 5 FGDs for FCHV, inclusive of 32 participants. Caregivers (all women) participated in 3 FGDs (n=18 participants). Data for the ranking task were also collected from the health volunteer and caregiver focus groups (n=50). In addition, 10 individual key informant interviews were conducted with FCHV, and 9 individual key informant interviews were conducted with caregivers. FCHV and caregivers were selected in Sankhu, Manamaiju, and Phutung. FCHV were identified from the roster of local government health facilities, where they report activities monthly. Every FCHV from 2 health facilities was selected for the study. The caregivers were selected based on referrals from FCHVs.

### Data Analysis

The audio recordings of FGDs and interviews were translated into English. The translated transcripts were loaded in NVivo version 12 (QSR International Pvt Ltd) for qualitative data analysis [46]. A framework coding analysis approach was used [47]. A priori codes included the devices (continuous audio recording, continuous video recording, environmental sensor, episodic audio recording, episodic video recording, proximity beacon, and wearable beacon) and device attributes initially used for the anchoring vignettes (acceptability, confidentiality, noninterference, safety, and utility or benefit). Additional a priori codes included themes from the qualitative interviews (c*ommunity health activities*: child development and behavior, child health program, community health worker activities, and information collection and *technology readiness:* mobile phone usage and other technology usage). Using the a priori codes, 2 coders (authors BAK, who was familiar with the Nepal context, and KV, who was familiar with the South Africa context) read and coded the same 4 transcripts (1 FGD from South Africa, 1 interview from South Africa, 1 FGD from Nepal, and 1 interview from Nepal).

The coders then used coder comparison within NVivo to determine areas of common versus discrepant coding. This was used to redefine the codes where needed. Additional code themes also emerged. These included *feasibility* and *health risks and injury* under attributes. Feasibility was added to address comments regarding whether the tools could be used but did not address acceptability or other attributes. Health risks was added to distinguish safety as an area where a person may be endangered by using the device through theft or assault versus health risks such as radiation or other perceived health consequences. Under community health activities, codes for *context of community and facilities* and *family and caregiver behaviors* were added. Under technology readiness, *barriers to technology use* and *facilitators of technology use* were added. All referenced quotations are provided in a supplemental file (see Multimedia Appendix 11).

The 2 coders then coded 4 new transcripts with the same breakdown by country and qualitative type to establish interrater reliability. The 2 authors achieved 0.80 interrater reliability. Subsequently, the coders reviewed half of the remaining qualitative dataset. Additional information on the qualitative process is available in Multimedia Appendix 12, using the consolidated criteria for reporting qualitative research framework [48].

Statistical analysis was performed on the card sort data for South Africa and Nepal datasets using median and interquartile range with inference testing using the Wilcoxon Rank Test. This approach was selected because the data elicited through the ranking tasks were nonparametric in distribution. Statistical analyses were performed with Statistical Package for Social Sciences version 24 [49].

### Ethical Approval

Ethical approval for the study was provided by the HSRC Research Ethics Committee in South Africa (REC6/18/05/16) and the Nepal Health Research Council (#241/2016) in Nepal. In addition, Duke University (Pro00074454) provided ethical approval for data analysis by US-based team members working with deidentified data from the sites. Participants in both sites completed written consents forms, which were also read to participants by research assistants because of low literacy rates among some groups in the study regions.

## Results

### Process Development for Qualitative Cultural Assessment of Passive Data Collection Technology

The QualCAPTD process was first conducted in South Africa (see Textbox 1). The procedure followed the steps as described in the Methods section above. During FGDs, the first part of the discussion was about child development, child health, and caregiver-child interaction. Then, the group watched all the videos in sequence; after watching all videos, they received paper forms to rate the devices on the attributes using the anchoring vignettes, and then, they ranked the devices on all of the attributes. After turning in the forms, the participants discussed the devices. The ratings with anchoring vignettes and ranking task evoked questions from participants, which suggested that when completing the forms, there may have been confusion about what the devices can and cannot do. Therefore, the procedure was modified for use in Nepal.

In Nepal, the first part of the FGD involved a similar discussion about child health and development and caregiver-child interactions. However, to increase understanding of the devices and clarity when discussing, rating, and ranking, we modified the procedure from the South Africa approach. Instead of showing all videos at once followed by completion of all rating and ranking, in Nepal, each video was shown with a break in between for rating and discussion, then ranking was done at the end after all videos had been shown, discussed, and rated (see Textbox 2). The Nepal procedure for devices went as follows: first, show one video; second, ask the group to discuss what was seen in the video and have open question and reflection; and third, introduce the rating on attributes with anchoring vignettes, the rating is completed as a group with each scoring discussed, nothing is written down by the participants, but the facilitator records the group consensus for device and attribute. Then, the group proceeds to the next video and rating discussion. After all videos have been shown, rated, and discussed, the participants are given the picture cards of the devices and asked to rank them according to each of the attributes. This is done individually by participants, and the scores are recorded for analysis. The next section describes the analyses of the ranking tasks in the 2 countries.

### Device Ranking

**Figure 5:**
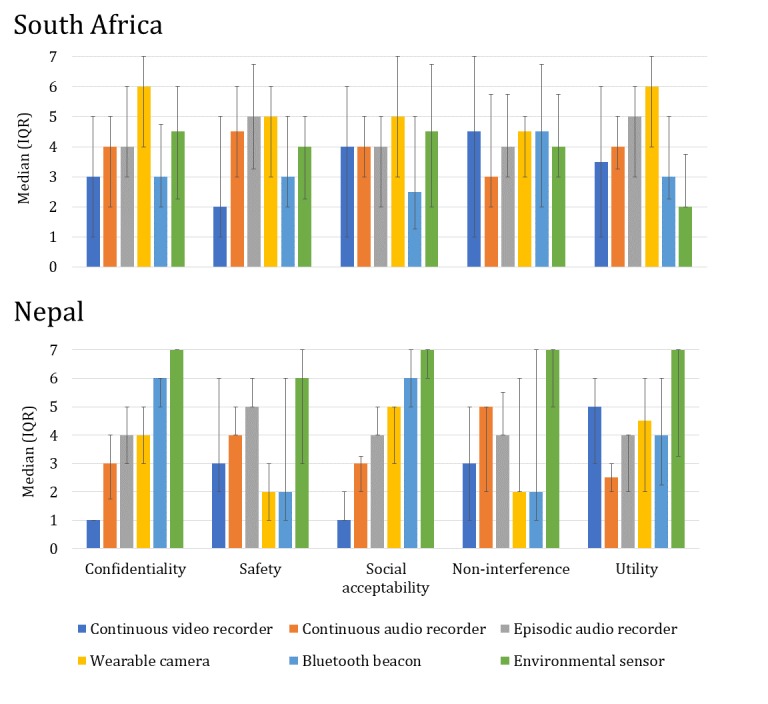
Card sort ranking results for attribute domains by country and device. IQR: interquartile range. South Africa (n=28); Nepal (n=50). Chi-square, Friedman test for ranking comparisons: South Africa: confidentiality (p=0.01), safety (p=0.01), social acceptability (p=0.17), non-interference (p=0.80), utility (p<0.001); Nepal: confidentiality (p<0.001), safety (p<0.001), social acceptability (p<0.001), non-interference (p<0.001), utility (p<0.001).

For the 88 focus group participants, ranking data were collected for all 7 data collection approaches (devices) across our 5 domains of inquiry (see Figure 5). In South Africa, 28 of the 38 participants’ data were usable for the ranking analysis, and there were significant ranking differences for the utility attribute (n=28, interrelated samples, chi-square, Friedman test, and *P*<.001). In South Africa, differences were significant only at the *P*<.05 level for confidentiality, safety, and noninterference. Rank orders were not significantly different for social acceptability in South Africa. The device rankings were significantly different across all 5 attributes for the Nepal sample (n=50, interrelated samples, chi-square, Friedman test, and *P*<.001).

### Qualitative Discussion Results

Qualitative findings from the video-centered (step 2) and anchoring vignette discussions (n=88) and interviews (n=26; step 3) were analyzed by domain and device. As there may have been confusion about the devices when ranked and rated individually by South African participants, the qualitative group discussions provided below may provide a better reflection of group attitudes and preferences.

#### Domain 1: Confidentiality

Regarding confidentiality, in South Africa, the wearable time-lapse camera and Bluetooth beacon were considered confidential in the qualitative discussions. In Nepal, the home-based environmental sensor was ranked the most confidential, followed by the Bluetooth proximity beacon. The home-based continuous video stream was the least confidential. In both countries, continuous audio recording was a concern because of capturing yelling at children and husbands yelling at the participants. In addition, if a husband knew that his wife had been recorded throughout the day, he may ask to review the audio recording. Mothers-in-law in Nepal were also considered to be interested in hearing the recordings, then spreading information to others in the community. Participants used the Nepali idiom, *ek kaan, dui kaan, maidan*, roughly translatable as “one ear, then two ears, then everywhere.”

**Pilot procedure for Qualitative Cultural Assessment of Passive Data collection Technology through focus group discussions in South Africa.**

Pilot procedure for Qualitative Cultural Assessment of Passive sensing Data Technology focus group discussions in South Africa:

- *Step a*. Group discussion about child health, child development, and caregiver-child interactions.
- *Step b*. Participants were shown all of the videos demonstrating the devices. No discussion was held between videos.
- *Step c*. Participants are given attribute rating forms with anchoring vignettes and device ranking; participants complete the forms independently for all devices. Participants are required to have sufficient literacy to read the forms and write answers.
- *Step d*. Participants return forms and have group discussion about devices.

**Refined procedure for Qualitative Cultural Assessment of Passive Data collection Technology through focus group discussions in Nepal.**

Refined procedure for Qualitative Cultural Assessment of Passive sensing Data Technology focus group discussions in Nepal.

- *Step a*. Group discussion about child health, child development, and caregiver-child interactions.
- *Step b*. The first video is shown.
- *Step c*. The group has an open discussion about what was seen on the video and can ask questions about the devices.
- *Step d*. As a group, the participants have a facilitated discussion to rate the device on each attribute using the anchoring vignettes. The group produces a consensus rating score for each attribute.
- *Step e*. Steps *b* through *d* are repeated for the remaining devices.
- *Step f*. Each participant is individually given a series of cards representing each device. They are asked to rank the cards for each attribute. No literacy skills are required, and each participant produces her own ranking.

##### Device 1: Video Recorder

Community health volunteers were concerned that mothers and their families would not allow the continuous video recording in the home because the family members would be afraid that others would disclose their confidential information (see Multimedia Appendix 11, qualitative code reference: CONF_CVR_Q01). Codes in parenthesis correspond to the qualitative reference number for the quotes provided in Multimedia Appendix 11. Respondents in South Africa reported that husbands may assume information will be shared with the police (CONF_CVR_Q04). Some women felt that the home-based video recording would actually put them in greater danger (CONF_CVR_Q05). Others wanted the camera to only be in the child’s room (CONF_CVR_Q06). A health worker in South Africa explained that she would be like community members in her community who would cover up the camera and not use it (CONF_CVR_Q07). Caregivers in Nepal were concerned about continuous video recording catching images of interpersonal violence (CONF_CVR_Q02). Some caregivers expressed that persons with bad behavior would not want others to know about this (CONF_CVR_Q03). Some caregivers and community health workers in Nepal explained that if families understood that only health workers used the information, then there would not be confidentiality problems (CONF_CVR_Q08). The health workers echoed this (CONF_CVR_Q09).

##### Device 2: Audio Recorder

Similar to the attitudes toward continuous video recording, participants were concerned that continuous audio recording would breach confidentiality (CONF_CAR_Q01, Q02, Q03). Caregivers expressed concern that having the continuous audio recording would incite arguments in the home. Some caregivers felt that the audio recording would have a negative impact over time by reducing the amount of emotional expression and by hearing negative things from others (CONF_CAR_Q04).

##### Device 3: Wearable Camera

Caregivers and health workers described their concerns about confidentiality when children wear the first-person time-lapse camera that takes photographs at preset intervals, such as every 30 seconds. With the child moving around the home and community while wearing the time-lapse camera, there was concern among community health workers in South Africa that they would capture nudity, drug use, and criminal activity (CONF_CWC_Q04). One concern was that the child would enter a room when the parents were being intimate and record this in the photographs. Caregivers did point out, however, that the child wearing a time-lapse camera would capture less compromising information than the continuous video recording (CONF_CWC_Q01). Similar to the continuous home video recording, South African health workers were concerned that the time-lapse camera worn by the child would capture images of domestic violence. The South African health worker also recommended GPS coordinates as an alternative data point that would be less of an invasion of privacy (CONF_CWC_Q02). Similar to the recommendations of other health workers, 1 Nepali community health volunteer emphasized the need for education of families about the child wearing the time-lapse camera and for all of the devices in general, which would assuage some concerns about confidentiality (CONF_CWC_Q03).

##### Devices 4 and 5: Bluetooth Beacon and Environmental Sensor

There were no confidentiality concerns about Bluetooth proximity beacons or environmental sensors.

#### Domain 2: Safety

##### Device 1: Video Recorder

Most discussions of safety focused on fear of theft. In South Africa, the home-based continuous stream video was considered the least safe, and no device was clearly ranked as the safest. Caregivers in Nepal reported that others could come into the house and steal the video camera (SAFE_CVR_Q01). In Nepal, where the respondents had recently experienced a major earthquake and were living in temporary housing, caregivers felt that they did not have a place to store the technology (SAFE_CVR_Q02). There were also concerns that the children could break the home video recorders (SAFE_CVR_Q03).

##### Device 2: Audio Recorder

There were no safety concerns.

##### Device 3: Wearable Camera

There was fear that children could break the wearable time-lapse camera that they would be wearing (SAFE_CWC_Q01). As the child wears the device, there was fear that others may steal the device from the children when the child is outside the home (SAFE_CWC_Q02, Q03). Other caregivers had similar concerns that the child would break the device or it would be stolen (SAFE_CWC_Q04). Program staff in South African health organizations had concerns that children would be mugged and the device stolen (SAFE_CWC_Q06). Similarly, in Nepal, the wearable camera was considered the least safe because the device could be stolen (SAFE_CWC_Q05).

##### Device 4: Bluetooth Beacon

The Bluetooth proximity beacon was considered a safety risk because it could be stolen from the children. In the qualitative interviews, the caregivers reported that they were afraid the Bluetooth beacon would be stolen from their children. Another safety concern in the qualitative interviews was that children—especially boys—would break the devices.

##### Device 5: Environmental Sensor

For safety, in Nepal, the environmental sensor was considered the safest. In subgroup comparisons in Nepal, community health workers rated the environmental sensor as less safe than how the caregivers rated it. In qualitative interviews, the community health workers reported that others may steal the environmental sensors to use in their own homes, whereas caregivers were not concerned that community members would steal the environmental sensors from one another.

#### Domain 3: Acceptability

There were no differences for social acceptability in the ranking task in South Africa sample, but the qualitative discussion suggests group preferences as described below. In Nepal, the environmental sensor and Bluetooth proximity beacon were the highest ranked and the home-based video the least acceptable. The Nepali respondents highlighted that the head of household and mother-in-law would need to consent to the using of the sensing devices and that they were least likely to accept the video recording in the home. Mothers-in-law often dictate the behavior of other women in the home, and not seeking support from mothers-in-law risk verbal and physical violence from the mothers-in-law. Respondents felt that head-of-household and mothers-in-law would support the environmental sensor because it would provide useful information for the household air quality, which would impact everyone’s health. Heads of household would also find the proximity beacon acceptable because it would not provide sensitive information about the family.

##### Device 1: Video Recorder

To use the devices in the home, participants in both countries reported that permission from the husband would be needed. In South Africa, the male head-of-household would need to grant permission (ACPT_CVR_Q04). An experienced health organization worker in South Africa pointed out that the most vulnerable children, for example, those being sexually abused, would be least likely to have the male head of household give permission (ACPT_CVR_Q05). To make it acceptable, it was told that the benefit would need to be explained. South African community health workers said that preventing violence against children would encourage families to accept the devices (ACPT_CVR_Q06). In Nepal, permission from mothers-in-law was also important (ACPT_CVR_Q01, Q02). Community health volunteers in Nepal stated that mothers-in-law who perform a majority of the child care may not consent because they would be criticized by the family members (ACPT_CVR_Q03).

##### Device 2: Audio Recorder

A South African health organization worker pointed out that getting families to consent to using an episodic audio recorder—or any of the devices—requires the same rapport building and long-term relationships that are needed for any health research activities (ACPT_EAR_Q02). Caregivers and health workers explained that all adult household members would need to consent for the continuous audio recording. Moreover, 1 health worker said that even adolescents would need to provide permission (ACPT_CAR_Q01). Caregivers in Nepal recommended getting permission from all adult household members before using the episodic audio recorder (ACPT_EAR_Q01).

##### Device 3: Wearable Camera

There were limited concerns about being able to obtain husbands and mothers-in-laws’ consent for using the child’s time-lapse camera in Nepal (ACPT_CWC_Q01). A community health worker, based on her expectations of gender and power in the community, stated that the child’s father’s permission would be needed (ACPT_CWC_Q02). Community health workers in Nepal were concerned about what would happen if the device was brought to school (ACPT_CWC_Q03). In addition, 1 community health volunteer in Nepal was concerned that it reflects negatively upon parents to send their children with the time-lapse camera (ACPT_CWC_Q04). This health worker continued to say that the child’s peers would not find it acceptable, and eventually, the child would be socially ostracized (ACPT_CWC_Q05).

##### Device 4: Bluetooth Beacon

In both countries, there were no concerns about the permission needed for using the beacons (ACPT_PRX_Q01, Q02).

##### Device 5: Environmental Sensor

Participants in both countries did not express concern about getting husbands or mothers-in-law consent for the environmental sensor, which they considered low risk. In Nepal, the community health volunteers compared installing a mobile sensor in the home with conducting the polio vaccination program. As with the polio program, consent from the mother would be adequate to deploy the sensor (ACPT_ENV_Q01). However, the males would be harder to convince than females, with the women in the family more likely to see the advantages of the devices for their children’s health (ACPT_ENV_Q02).

#### Domain 4: Noninterference

No single device stood out in South Africa as not interfering, but the qualitative discussion suggested that the continuous stream audio recorder was the most interfering. In Nepal, the environmental sensor was ranked highest, and the wearable time-lapse camera and Bluetooth proximity beacon were the most interfering. The main concern of caregivers was that the devices would be interfering because they would be attached to the children’s clothing. In subgroup comparisons in Nepal, the caregivers considered the continuous audio recording as interfering in daily activities, whereas they did not consider the wearable time-lapse camera or Bluetooth beacon as disruptive as community health workers did.

##### Device 3: Wearable Camera

The main concern in terms of disruptiveness of the device was related to the child’s time-lapse camera, which could interfere with the child’s activities, as noted by respondents from both countries (INTF_CWC_Q01-Q05).

#### Domain 5: Utility

In South Africa, the wearable time-lapse camera was the most useful and the environmental sensor was the least useful. In Nepal, the environmental sensor was ranked as the most useful for child health and well-being. Both health workers and caregivers were very concerned about pollution and the impact on child health. They wanted to be able to monitor how much exposure the children had to pollution, given the frequency with which their children were getting respiratory infections. The continuous stream audio recorder was ranked the least useful. In both countries, both caregivers and community health workers were interested in observing children’s lives around and outside the home. The wearable time-lapse camera was seen as a way to understand more about what children were doing. Parents were interested also because they reported that grandparents assumed the majority of child care responsibilities and they interacted with the children primarily in the morning and evening, so the wearable time-lapse camera would allow them to learn more about their children’s lives—especially for preverbal children. In Nepal, in subgroup comparisons, the caregivers ranked the wearable time-lapse camera as more useful than the health workers did. Another aspect of wearable time-lapse camera utility was as a teaching tool. Both caregivers and health workers reported that having more information about their own behavior and interaction with children could help them to improve how they care for their children. Some commented that hearing themselves yell at children may help them reduce the behavior. Regarding utility, both caregivers and health workers reported that they did not see the technology as a substitute for face-to-face interaction. They preferred face-to-face interaction for getting health information and engaging with community health workers in the home.

##### Device 1: Video Recorder

Caregivers described the perceived benefit of being able to observe children’s physical activity in the home as well as how other adults behave toward children and if there is any abuse occurring in the home (UTLT_CVR_01-CVR04). A South African health worker pointed out that the video could be proof of abuse even when the family denies it. This is especially important for preventing rape and associated potential HIV transmission (UTLT_CVR_05-06). The video was seen as a way to check on their children’s health (UTLT_CVR_07). South African health workers also raised the possibility that the video would capture sexual abuse in the home (UTLT_CVR_Q10). The community health workers in South Africa described how the video could help them better educate mothers and other caregivers about addressing child care needs while also maintaining other household responsibilities (UTLT_CVR_Q11). The video could also lead to suggestions about sleeping arrangements for children in the household (UTLT_CVR_Q12, Q13). Tracking sleep of school children through the video was important for a South African health worker (UTLT_CVR_Q14). FCHVs in Nepal reported the advantage for their home health work in being able to observe the daily activities (UTLT_CVR_Q08, Q09). In Nepal, the caregivers and FCHVs also perceived home-based continuous video as a monitoring tool to watch everyday risks the children might face such as falls and playing in dirt, and they could use observations from the video to correct negative behaviors of their child.

##### Device 2: Audio Recorder

In South Africa, community health workers reported that hearing parent-child communication as well as harsh communication in the household would be helpful for their work (UTLT_CAR_Q07). Caregivers in Nepal commented on the benefit to audio recording above and beyond that, which is obtained through video only (UTLT_CAR_01). Regarding health condition, the caregivers mentioned the audio from children with cough and cold symptoms, although it was debated whether video or audio would be preferable for this (UTLT_CAR_Q02, Q03). The audio recordings were also seen as advantageous for behavioral change among caregivers and other family members (UTLT_CAR_Q04). Some community health workers in Nepal commented that having domestic arguments recorded could facilitate their health promotion work and talk about communication in the household (UTLT_CAR_Q05, Q06). A caregiver in Nepal thought it would be helpful for older children (UTLT_CAR_Q08, Q09). The episodic audio recorder was perceived by health workers to help understand the auditory environment in which the child is living and its potential impact on child development, learning, and behavior (UTLT_EAR_01). The audio snippets could be used to determine the types of interactions caregivers and children are having (UTLT_EAR_02). Caregivers also felt that they could learn more to make sure that the child was getting the appropriate exposure to positive auditory stimulation, including language exposure for preverbal children (UTLT_EAR_Q03, Q04). The audio snippets could also be used to identify potential abuse (UTLT_EAR_Q05).

##### Device 3: Wearable Camera

Community health workers preferred the child’s wearable time-lapse camera over the audio recordings because of the information it would provide on activities (UTLT_CWC_01). A South Africa caregiver preferred it over audio because it would not capture private speech or yelling at the child (UTLT_CWC_Q02). Compared with the continuous video recorder in the home, caregivers liked the child’s wearable time-lapse camera because it provides information from wherever the child went rather than being restricted to the home (UTLT_CWC_Q03, Q04). Health workers in South Africa liked how the child’s wearable time-lapse camera would provide information about the child’s diet and nutritional intake (UTLT_CWC_Q08, Q09). Some caregivers perceived that the wearable time-lapse camera would capture images of a kidnapper if the child were abducted (UTLT_CWC_Q10). In Nepal, caregivers felt that the utility of continuous recording was limited because children spend time in many different rooms. Therefore, the wearable time-lapse camera was more beneficial (UTLT_CWC_Q05). In contrast, compared with continuous video recorder, 1 caregiver in Nepal felt that the child’s wearable time-lapse camera would not capture as much useful information as the continuous video in the home (UTLT_CWC_Q06). Similarly, another caregiver preferred the continuous video to the child time-lapse photography (UTLT_CWC_Q07).

##### Device 4: Bluetooth Beacon

In South Africa, a health worker appreciated the concept of tracking mother and child time together based on the rationale that bonding is important for child development (UTLT_PRX_01). Similarly, caregivers in Nepal appeared to value time together with children, especially mother and child; therefore, something could be gained from tracking that with the Bluetooth proximity beacon (UTLT_PRX_Q02, Q03). In general, in both sites, the caregivers conceptualized the Bluetooth beacon as a tracking device that would allow them to find the child if mother and child were separated or if the child was kidnapped (UTLT_PRX_Q04, Q05).

##### Device 5: Environmental Sensor

The environmental sensor was considered to have major utility by Nepali participants, given the high rates of respiratory illness morbidity and mortality. Caregivers reported that they could use the information to adjust when they go outdoors and when to wear a mask (UTLT_ENV_Q01, Q02, Q03). With major campaigns to change cookstoves and indoor cooking, the environmental sensor was considered useful to detect air quality related to cooking (UTLT_ENV_Q04). Community health workers in Nepal were interested to use this information to educate families about air quality within different rooms in the house (UTLT_ENV_Q05).

## Discussion

### Principal Findings

#### Process for Qualitative Cultural Assessment of Passive Data Collection Technology

We found that the QualCAPDT procedure could be implemented in low-resource settings where populations would benefit from passive sensing data for health and development interventions. The procedure produced both quantitative and qualitative results that could be used to select devices for piloting. This is helpful to determine devices that would be culturally appropriate. The process also reveals what concerns would need to be addressed in the development and use of such devices for passive sensing data collection. The procedure can be feasibly implemented in communities with low literacy rates. We piloted the procedure in South Africa but made further modifications when implemented in Nepal to optimize feasibility for use with illiterate caregivers and to increase the understanding about devices for the participants when completing the ratings and rankings.

In South Africa, upon original administration of the anchoring vignettes, participants completed all the vignettes after watching all the videos. In contrast, in Nepal, the anchoring vignettes were completed after each individual video was shown. We made this modification because in South Africa, the participants had confusion about the devices, and by the time they watched all videos, there was some difficulty remembering the different features of the devices when rating them. The approach of discussing each video immediately after presentation allows for immediate recall of the features. This also allows the group to develop areas of consideration that can be incorporated when watching the subsequent videos. The goal of this process is for persons unfamiliar with the different technologies to discuss potential application in their local context; therefore, it was important to give them time to consider each device individually. This likely facilitated a clearer group understanding of the devices before proceeding to the ranking activity in step 4. Moreover, the modified process used in Nepal allows for low literacy and illiterate populations to participate. In the modified procedure for Nepal, no reading or writing was required and the card ranking task only required sorting pictures of the devices. Going forward, we recommend the Nepal approach of watching each video and discussing as a group to produce more well-formed and well-considered ratings. This likely contributed to the clearer ranking outcomes observed in Nepal compared with South Africa.

Misperceptions generally improved from the South Africa to Nepal implementation. However, there were still misunderstandings, which highlights the need to do more to clarify what devices *can do* and *cannot do*. This clarification is important to get accurate ratings. In South Africa, participants thought proximity beacons could locate kidnapped or lost children. In both countries, participants thought home-based or wearable cameras would allow for surveillance of sexual predators. Participants in South Africa thought proximity beacons could locate children if they were kidnapped, and this may have led to higher expectations of benefit. In Nepal, the home-based video was thought to be similar to a closed-captioned television security camera and, therefore, may have led to higher ratings on the utility ranking. These perceptions would need to be addressed if such technologies were implemented to manage expectations of participants in health programs. This also illustrates why a method such as QualCAPDT is helpful to illuminate potential misinterpretations.

#### Recommendations for Implementing Qualitative Cultural Assessment of Passive Data Collection Technology

Given the development and piloting of QualCAPDT in South Africa and Nepal, we have the following recommendations for using the procedure in other settings (see also Table 2):

- *Step 1: Use qualitative interviews to assess the landscape of current needs for beneficiaries who would later use the technology*. This may be health workers, parents, educators, or others with a home-based component of their programs. The formative qualitative work should determine current technology use and potentials for new devices and/or data collection approaches with existing devices. The goal of Step 1 is to have contextual information to better inform device selection and data collection strategies as well as the relevant device attributes that will need to be assessed (eg, protecting confidentiality, minimizing interference with other activities, and perceived benefit to end users).
- *Step 2: Select the attributes that will be assessed in the QualCAPDT process.* In our study, our a priori attributes were confidentiality, acceptability, safety, nondisruptiveness, and perceived utility. However, we found that adding an attribute on health impacts during the qualitative analysis was helpful to split the safety construct into issues related to theft and risk of violence versus impact of the device on health (eg, through perceived radiation exposure). In future studies, the attributes should reflect the characteristics of devices and needs of the projects. Buenaflor et al’s 6 factors found to influence adoption (supporting fundamental human needs, cognitive load, social factors, physical aspects, participant demographics, and technical expertise of the user) [22] can be helpful to determine attributes. We caution developers that the wording of the attributes should be easily intelligible to beneficiaries in low-resource settings.
- *Step 3: Develop a range of candidate technologies and data collection approaches to demonstrate in videos.* Some devices or data collection approaches may be excluded at this stage based on the formative qualitative work in Step 1 and consideration of attributes from Step 2.
- *Step 4: Produce brief videos to demonstrate the technology.* This should be short (fewer than 5 min) so that multiple can be shown in FGDs with sufficient time. The setting of the videos should as closely approximate the context of end users. We found it helpful to illustrate what the technology is and how it is used in the home, what type of data are being collected, and how those data will be potentially used by health workers. On the basis of the experiences in South Africa and Nepal, it is also helpful to describe the limitations of the devices, for example, a tool such as a passive Bluetooth beacon has a limited range that would not allow tracking a child lost away from home or abducted.
- *Step 5: Develop anchoring vignettes to ask about the attributes for each device.* These anchoring vignettes should describe contrasting preferences for each attribute, for example, Maya does not want the device in her home because her family would be concerned about privacy, but Asha thinks her family would agree to allow it. Then, ask “Are the women in your community more like Maya or Asha?” and “Why?” It is helpful to create visuals of persons representing the different anchors so that participants can point to how close their community is to either of the anchors (see Figure 3).
- *Step 6: Create picture cards with the different technology that can be used for the ranking tasks.* This is helpful so that participants do not need any literacy skills to participate in the card sorting.
- *Step 7: Pilot the process with approximately 2 FGDs.* This is helpful if the team has not previously used structured elicitation tasks. The facilitators can practice their approach including explaining anchoring vignettes and card ranking. In addition, the participants may raise questions about what is demonstrated in the videos. From the questions, the facilitators may learn what else needs to be explained in the subsequent FGDs.
- *Step 8: Conduct FGDs with the full proceedings recorded for transcription, translation, and subsequent qualitative data analysis.* The FGD should begin with first discussing general issues separate from the technology but related to the theme of the future technology use. Then, the first video should be shown. This should be followed by a general discussion of the device and data collection in the video. After open discussion, the participants go through the anchoring vignettes as a group for each attribute. The facilitator records the group consensus for each attribute and reasons for that rating. Then, the next video is shown and steps repeated. After all videos are shown, discussed, and rated, the participants are given the pictorial cards of the devices, and they go through the ranking process. The rankings for each individual participant are documented.
- *Step 9: Qualitatively analyze the FGDs.* The qualitative analysis should be conducted first to prevent bias introduced by reviewing the ranking scores. Although the a priori attributes should be used, there should also be an opportunity to introduce new attributes (eg, adding “health” in our experience). Then, quantitative data can be analyzed using techniques such as Wilcoxon Sign-Rank test. The data from both should then be synthesized and summarized using recommended approaches for mixed-methods research [50,51].
- *Step 10: Conduct supplementary interviews.* Although we conducted our supplemental interviews before the analysis, it is preferable to follow qualitative and mixed-methods research recommendations to share results with members of the target group to collect their interpretation of the findings [48]. This can be helpful to discuss issues and concerns that may not have arisen in the formative work. For example, concerns related to sexual violence against young children was not part of the planning of our project but arose in the FGDs and would need to be discussed with stakeholders before proceeding to final selection of the technology and data collection process.

Ultimately, this approach is helpful to prepare for future passive data collection. It will work best when there is adequate piloting and incorporation of user-centered design principles. This will increase the likelihood that the passive sensing data collection is acceptable, feasible, and beneficial to promote child health and development and caregiver-child relations.

#### Device Preferences

We found that in South Africa and Nepal, there was interest in observing the world from the child’s perspective through wearable cameras that could be placed on the children and then have the caregivers review the photographs. In terms of utility, caregivers were open to using the technology to help them gain insight into their behavior with their children and then find ways to improve such behavior. Health workers also felt that sensing data could help them better educate parents. As this was a primary goal of the sensing technology, it was reassuring that both caregivers and health workers were open to this aspect of technology use.

In Nepal, there was a consistent concern across the ranking, rating, and interviews that devices attached to the children could be easily stolen and that by being attached to clothing, they would interfere in daily life in some way. For safety, we had expected that concerns about technology making children sick in some manner or being a health risk. However, safety was understood in terms of risk of theft. Participants in Nepal felt that devices attached to children could easily be stolen and may put them at risk. There were also concerns about what devices could be stolen from the home.

**Table 2:** Recommended steps for Qualitative Cultural Assessment of Passive Data collection Technology.

Steps for Qualitative Cultural Assessment of Passive Data collection Technology	Description
Step 1: conduct formative interviews	Conduct interviews with beneficiary population about roles, responsibilities, and current technology use
Step 2: determine criteria/attributes	Determine criteria for potential technologies to be evaluated
Step 3: select candidate technologies	Select a range of candidate technologies to fulfill the needs of the target population
Step 4: develop videos	Write scripts explaining technologies and then produce videos to illustrate what technologies will do; also include descriptions of what the technologies will not be able to do
Step 5: develop anchoring vignettes	Develop anchoring vignettes for each technology based on identified criteria/attributes
Step 6: develop cards for ranking tasks	Develop visual illustrations of candidate technologies for ranking tasks to minimize literacy skills needed to recognize the technologies; consider using pictures from videos that participants will be shown
Step 7: pilot videos and refine (eg, add clarifications of “can do” vs “cannot do”)	Pilot test videos with beneficiary representatives to determine what is perceived about the use of technology; consider developing additional “can do” and “cannot do” explanations to modify the videos or to be used by the FGD^a^ facilitators, for example, proximity beacons cannot track location of children away from the home
Step 8: conduct FGDs	Conduct FGDs showing videos, include anchoring vignettes and card ranking, and allow for “can do” and “cannot do” discussion
Step 9: analyze qualitative data, then analyze quantitative data	First analyze data qualitatively using a priori themes based on attributes and allow for addition of new themes and attributes; then, quantitative analyze individual participant ranking scores of devices for each attribute using Wilcoxon Rank or other appropriate statistical tests
Step 10: conduct supplemental interviews	Conduct supplemental interviews to obtain feedback from stakeholders and collect their interpretation of findings from the qualitative data and ranking statistics

^a^FGDs: focus group discussions.

A major issue for consent was that the majority of household members would need to be involved in the consenting process to allow for use of passive data collection technologies. In Nepal, social acceptability was predominantly dependent upon what the head-of-household and mother-in-law would find permissible and helpful. Most women felt that the environmental sensor and proximity beacon would be approved by these authority figures.

In Nepal, it was striking how much the environmental sensor was consistently the highest ranked device across all attributes. Both caregivers and health workers strongly desired to have information about pollution and air quality. As with other cities in South Asia and East Asia, there is increasing public health awareness about the impact of pollution. Using a rating procedure by residents, Kathmandu is ranked as the fifth highest city in the world in the Pollution Index Rate, as a reference, Delhi is 14 and Beijing is 23 [52]. In addition, children—and adults—have high rates of respiratory infection morbidity and mortality, with pneumonia being the leading cause of death for children under 5 years [53]. In addition, the environmental sensor was seen as preserving confidentiality, having a low risk of theft, being acceptable to heads-of-household and mothers-in-law, and not interfering with other activities in daily life. This suggests that future studies that wanted to explore air quality and child health at the household level would have high buy-in from participants and family members. This type of research could also support advocacy of families to politicians about addressing environmental health.

In South Africa, health workers reported that they were already using phones in the home, so that may help to have them transition to more mobile technology to improve their work. Although environmental influences on child development stood out in the Nepal findings, a concern in the South Africa study was the goal to protect children from sexual violence. They were interested in ways in which the sensing technology could collect information on sexual perpetrators including relatives and that the technology could be used to prevent perpetration of sexual violence because community members would be aware of the data collection.

#### Contributions of the Qualitative Cultural Assessment of Passive Data Collection Technology Procedure to Future Passive Sensing Data Research

The methods that we used for this study incorporate techniques from social sciences and cultural anthropology that are useful for conducting mHealth studies in similar settings in the future. Use of technology in health-related research and intervention is already an integral part in developed countries and has been growing in pace in developing countries such as Nepal [54-56]. However, adaptation and contextualization of these technologies in these settings itself requires a rigorous process. These settings have populations with limited or no exposure to complex technologies, and the available infrastructures are also limited. Explaining technologies to potential beneficiaries through videos instead of traditional didactic methods helps in better understanding as they are more likely to grasp its concept when watching how it works. The use of anchoring vignette with a third-person character simplifies the comparative process and minimizes the social desirability biases [44], that is, the responses are external to the individual and participants are not bound to present themselves as someone agreeable, which is very important in some cultural settings. Asking them about their thoughts embedded in fictional characters will lead to more honest and rich answers in relation to future end-user behavior. These processes will help researchers and implementers in selecting technologies that the community understands, acknowledges, approves, and considers useful.

During the last 5 to 10 years, there has been substantial research energy invested into the field of mHealth [6]. Immediate and plentiful opportunity existed to leverage and embed mobile phones into the global health system. The mHealth research agenda was aided by the fact the mobile phones were ubiquitous and easy to use and understand. For example, receiving a health system–generated medication reminder via short message service felt familiar and required no further explanation. However, as we move beyond the phone and begin to harness embedded sensors and invisible technologies that are not well understood, better methods will be required to facilitate full understanding between the researcher and the participant. The QualCAPDT process offers 1 approach to ensuring that all technology introduced into the home to support maternal and child health and development is not done so at the cost of full consent and understanding of those providing these rich yet intrusive data. Ultimately, the QualCAPDT procedure can be used to identify devices and passive data collection approaches to further advance tailored messaging to caregivers and health workers [57] for the goal of improved physical, mental, and developmental well-being of children and their caregivers.

### Limitations

One limitation is that the development of the QualCAPDT procedure evolved over time and modifications were made both within and between countries over time. Therefore, statistical comparisons between rankings in South Africa and Nepal are not appropriate. Another limitation was that we did not specify different developmental stages of children when considering responses. The respondents likely would have had different qualitative responses and rankings if they considered the child health and development needs of infant versus adolescent. For the most part, the respondents appeared to consider toddlers and early childhood in making their responses.

### Conclusions

In settings where populations have limited prior exposure to passive data collection, methods are needed to determine what may be acceptable and have high perceived local value. Through the use of videos to demonstrate local implementation of technologies and subsequent structured elicitation tasks, we assessed norms and preferences in settings with high public health need for child development and caregiver mental health interventions. Future steps in our research will be using the current findings to develop and pilot passive data collection with children and caregivers. The future research will illuminate how well the qualitative elicitation method captured key community expectations and concerns. Ultimately, a structured qualitative elicitation procedure is an important method for selecting new research technologies.

## Appendix

Multimedia Appendix 1 Video recorder demonstration video for South Africa.

Multimedia Appendix 2 Video recorder demonstration video for Nepal.

Multimedia Appendix 3 Audio recorder demonstration video for South Africa.

Multimedia Appendix 4 Audio recorder demonstration video for Nepal.

Multimedia Appendix 5 Wearable camera demonstration video for South Africa.

Multimedia Appendix 6 Wearable camera demonstration video for Nepal.

Multimedia Appendix 7 Bluetooth beacon demonstration video for South Africa.

Multimedia Appendix 8 Bluetooth beacon demonstration video for Nepal.

Multimedia Appendix 9 Environmental sensor demonstration video for South Africa.

Multimedia Appendix 10 Environmental sensor demonstration video for Nepal.

Multimedia Appendix 11 Qualitative data quotations.

Multimedia Appendix 12 Consolidated criteria for reporting qualitative research (COREQ) items.

## Abbreviations

FCHVs: female community health volunteers
FGDs: focus group discussions
GPS: Global Positioning System
HSRC: Human Sciences Research Council
LENA: language environment analysis
LMIC: low- and middle-income countries
mHealth: mobile health
QualCAPDT: Qualitative Cultural Assessment of Passive Data collection Technology
RFID: radio-frequency identification devices
SDGs: Sustainable Development Goals
