# Supplementary material for: Procedures to Select Digital Sensing Technologies for Passive Data Collection With Children and Their Caregivers: Qualitative Cultural Assessment in South Africa and Nepal
Source: JMIR Pediatr Parent. 2019 Jan 16;2(1):e12366. doi: 10.2196/12366 (PMC6716492; doi:10.2196/12366)
Supplement: Multimedia Appendix 12 [file pediatrics_v2i1e12366_app12.pdf]

Supplemental File. Consolidated criteria for reporting qualitative research (COREQ) [48]

Item	Description
**Domain 1: Research team and reflexivity**	
1. Interviewer: which author(s) conducted interviews	KR, KV, and research assistants
2. Credentials: researcher's credentials	BAK, MD, PhD; SR, MA; KV, MS; KT, MPH; AB, BS; AvH, PhD
3. Author occupation at time of study	BAK, assistant professor; SR, research associate; KV, master's degree intern; KT, research assistant; AB, research associate; AvH, research director
4. Gender of interviewer(s)	Male and Female
5. Experience and training of authors	BAK, PhD in anthropology with training in qualitative methods, ethnography, and structured elicitation tasks, and 20 years of experience in qualitative and mixed-methods research; SR, five years of experience with qualitative research; KV, master's degree training in qualitative research; KT, MPH with coursework in qualitative research; AB, two years of experience in qualitative research; AvH, 10 years of experience in mixed-methods research.
6. Was a relationship with participants established prior to study commencement	South Africa: research participants had previously participated in other research projects through Human Sciences Research Council<br>Nepal: Participants were introduced to research team through community liaison who was a health worker and known to the study team
7. What did the participants know about the researchers?	South Africa: participants were aware the research team was associated with local public health research organization that was involved in research, care delivery, and making research-informed policy recommendations<br>Nepal: Participants were aware that research team had conducted prior mental health programs for adolescents affected by a recent earthquake
8. What characteristics were reported about the interviewer(s)?	Organizational affiliation
**Domain 2: Study Design**	
9. Methodological orientation underpinning the study?	Qualitative framework with anthropological structured elicitation tasks
10. How were participants selected?	107
11. How were participants approached?	107
12. What was the sample size?	107
13. How many people refused participation?	0
14. Where was the data collected	Sweetwaters, Kwa-Zulu Natal, South Africa<br>Lubhu and Sankhu, Kathmandu Valley, Nepal

Item	Description
15. Was anyone else present besides the participants and researchers?	No
16. What are the important characteristics of the sample?	Community health workers and caregivers
17. Were questions, prompts, guides provided by the authors? Was it piloted?	Guides were used; piloting was conducted
18. Were repeat interviews carried out?	No
19. Did the research use audio or visual recording	Yes, audio recordings were used
20. Were field notes made during the interview?	Yes, interviewers took field notes after the interview to record non-verbal expressions and any interruptions during the interview
21. What was the duration of the interview?	Interviews ranged from 30 minutes to 2 hours
22. How was data saturation ensured?	Review of field notes by research assistants and study supervisors
23. Were transcripts returned to participants for comment and/or correction	No
**Domain 3: Analysis and Findings**	
24. How many data coders coded the data?	Two coders were assigned to code the data
25. How was coding applied?	Coding was done in NVivo 12
26. Were themes identified in advance or derived from the data?	Codes were identified in advance and additional codes were derived from data
27. What software was used to manage and analyze data?	NVivo 12
28. Did participants provide feedback on the findings?	No
29. Were quotations presented to illustrate findings? Was each quotation identified?	Quotations are provided in supplemental file
30. Was there consistency between the data presented and the findings?	Quotations and data presented were compared with reported findings by authorship team
31. Were major themes clearly presented in the findings?	Themes are presented and linked to original quotations
32. Is there a description of diverse cases or discussion of minor themes?	Minor themes are presented descriptively within narrative descriptions
